# Measuring Hospital Performance Using Mortality Rates: An Alternative to the RAMR

**DOI:** 10.15171/ijhpm.2017.94

**Published:** 2017-08-12

**Authors:** Christine Pitocco, Thomas R. Sexton

**Affiliations:** College of Business, Stony Brook University, Stony Brook, NY, USA.

**Keywords:** Hospital Performance Measures, Mortality Rate, Risk Adjustment

## Abstract

**Background:** The risk-adjusted mortality rate (RAMR) is used widely by healthcare agencies to evaluate hospital performance. The RAMR is insensitive to case volume and requires a confidence interval for proper interpretation, which results in a hypothesis testing framework. Unfamiliarity with hypothesis testing can lead to erroneous interpretations by the public and other stakeholders. We argue that screening, rather than hypothesis testing, is more defensible. We propose an alternative to the RAMR that is based on sound statistical methodology, easier to understand and can be used in large-scale screening with no additional data requirements.

**Methods:** We use an upper-tail probability to screen for hospitals performing poorly and a lower-tail probability to screen for hospitals performing well. Confidence intervals and hypothesis tests are not needed to compute or interpret our measures. Moreover, unlike the RAMR, our measures are sensitive to the number of cases treated.

**Results:** To demonstrate our proposed methodology, we obtained data from the New York State Department of Health for 10 Inpatient Quality Indicators (IQIs) for the years 2009-2013. We find strong agreement between the upper tail probability (UTP) and the RAMR, supporting our contention that the UTP is a viable alternative to the RAMR.

**Conclusion:** We show that our method is simpler to implement than the RAMR and, with no need for a confidence interval, it is easier to interpret. Moreover, it will be available for all hospitals and all diseases/conditions regardless of patient volume.

## Background

Healthcare costs in the United States rose 5.3% in 2014 to $3.0 trillion, or over $9500 per capita, reaching 17.5% of gross domestic product (GDP),^1^ which has created recurring and urgent demands to reduce healthcare costs. Hospitals may look for ways to reduce cost by reducing the number of treatments, using less expensive interventions, or reducing length of stay, thereby risking quality degradation. Thus, the need to monitor provider performance has perhaps never been greater.

Comparing hospitals based on their outcome measures is a relatively common practice. Healthcare agencies often use hospital mortality rates within specific disease or condition categories to measure performance. Often state health departments and the Center for Medicare and Medicaid^2^ release these reports to the public. The Leapfrog Group^3^ and the Agency for Healthcare Research and Quality (AHRQ)^4^ contribute to these reports. The public as well as healthcare agencies look to these reports, which may be difficult to interpret, to find quality healthcare providers.

A proper comparison of mortality rates must include an adjustment for risk variation that “levels the playing field” so that a hospital’s evaluation is independent of the risk profile of its patients. Risk factors commonly include age, comorbidities, illness severity, and other patient and case characteristics. The most commonly used adjustment method is the risk-adjusted mortality rate (RAMR) defined later.

Hibbard et al^5^ found that hospitals whose RAMRs were made public were more likely to engage in quality improvements efforts than those who had a confidential quality report or no quality report at all. Baker^6^ showed that the 30-day mortality rate in New York State (NYS) after coronary artery bypass graft declined 41% between 1989 and 1992 after public reporting, and that published mortality rates impacted referral rates among cardiologists in NYS, thereby affecting provider market share and patient choice of healthcare provider. Schneider and Lieberman^7^ report that, when choosing health plans, employers use these “report cards” to identify high quality health plans at reasonable costs. Similarly, employees may select plans, doctors and hospitals using report card information about providers, quality score, price and accessibility. Moreover, a poorly rated provider may cease operations leaving the local area with a diminished supply of a critical healthcare service.

However, previous studies have questioned the use of the RAMR for this adjustment. Iezzoni^8^ shows that different methods can provide varying judgments. Thomas and Hofer^9^ questioned the accuracy of the RAMR as a valid indicator of a hospital’s quality performance, and conclude that “reports that measure quality using RAMRs misinform the public about hospital performance.” Dimick et al^10^ show that the RAMR may not be appropriate for all procedures, particularly when the sample size is small. Scott^11^ discusses the statistical difficulties associated with a rare outcome (death), the omission or difficulty of proper measurement of patient-related prognostic factors, and the weak and inconsistent correlation between hospital-wide RAMR and explicit quality indicators. He concludes the RAMR is a poor indicator of unsafe hospital care.

There are a number of ways a hospital’s performance is measured. Racz and Sedransk^12^ modeled risk-adjusted assessments utilizing Bayesian and frequentist indirect standardization methods, which they compared to the RAMR for “provider profiling.” These methodologies produced very similar results although they found markedly fewer outlying hospitals when the random-effects assumption was applied to the hospitals. The Leapfrog Group^3^ provides a hospital safety score that is generally a grading system that shows how safe a hospital is for patients. The purpose of the score is to provide a mechanism to consumers in which they can educate themselves about the safety of a facility they may be considering. The scores are publically reported and are available online. In addition, the Joint Commission’s ORYX program^13^ integrates performance measurement into their accreditation and quality improvement processes. The Center for Medicare and Medicaid Services (CMS)^2^ publicly reports process performance measures based on data collected by the Hospital Quality Alliance (HQA).^14^

In this paper, we will demonstrate that the RAMR is intrinsically a highly flawed performance measure and that, moreover, it is applied improperly. We will then propose an alternative to the RAMR, along with statistically appropriate implementation methods, that avoids all the problems listed above without requiring additional data. Our alternative measure has a clear and practical interpretation, is based on standard statistical theory and methods and, unlike the RAMR, can be applied regardless of the number of cases.

## Methods

We demonstrate our methodology using data from the NYS Department of Health (NYS DOH).^15^ We chose to test our model using NYS data for several reasons. NYS was among the first states to use the RAMR, applying it to cardiac surgeons and the hospitals in which they performed cardiac surgery starting in 1989. Kasprak^16^ cites the NYS DOH program as “the first program in the country to produce public data on outcomes for cardiac surgery and is the nation’s longest running program of its kind.” During the intervening 28 years, the NYS DOH has dramatically expanded its use of the RAMR to 24 inpatient quality indicators (IQIs). It also applies the methodology to complications and readmissions. NYS DOH makes its raw data readily available to the public through its web site.^15^ We believe that, if we can effect improvements in NYS, then other states are likely to follow.

### The Risk-Adjusted Mortality Rate

The RAMR attempts to account for the differing risk profiles of patients. It is standard practice to use a logistic regression model for a given procedure or illness to estimate each patient’s probability of death based on their patient and case characteristics. The mean and standard deviation of the number of deaths, and therefore the expected mortality rate (EMR) for a procedure or illness at a given hospital, is then estimated from these probabilities. The ratio of the observed mortality rate (OMR), which equals the number of deaths divided by the number of cases, to the EMR is then multiplied by the statewide observed mortality rate (SOMR) or national mortality rate (NMR) to obtain the RAMR.

Suppose that a hospital treats *n* patients with a particular diagnosis and that *X* of these patients die. Then the OMR = X/n. Define a variable called Death, which equals 1 if the patient died, or equals to 0 if the patient survived. Next, consider a logistic regression model that has Death as its dependent variable and a set of established mortality risk factors for that diagnosis as its independent variables. Suppose that this model estimates probabilities of death of *p*_1_, *p*_2_, …, *p*_n_, for the *n* patients. Then the hospital’s $EMR=\frac{1}{n}\sum_{i=1}^{n}p_{i}$ and its $RAMP=\left(\frac{OMR}{EMR}\right)\;SOMR$. Thus, RAMR < SOMR (alternatively, RAMR > SOMR) indicates better-than-expected (or worse-than-expected) performance.

According to Marang-van de Mheen and Shojania^17^ the hospital standardized mortality ratio ($HSMR=\frac{OMR}{EMR}$) is not a sufficient measure to inform patients and policy-makers as to whether the mortality risk is higher in a hospital compared to another. In addition, the HSMR may be affected by Simpson’s paradox. Although this may not cause direct harm to a patient, this may cause hospitals that need to address problems not to do so and may cause hospitals that actually do have problems not to address them.

### Shortcomings of the Risk-Adjusted Mortality Rate and its Application

We identify five shortcomings of the RAMR:

*The RAMR is a poor indicator of hospital performance*: We demonstrate that the RAMR needs to be augmented by additional information before a proper interpretation of its value is possible. Consider Ellenville Regional Hospital, located in New York State’s Hudson Valley. In 2013, they treated 42 patients with pneumonia, two of whom died. Therefore, their OMR was 2/42 = 4.76%. Based on the patient and case characteristics of their 42 patients, their EMR was 2.06%. Thus, their ratio OMR/EMR = 2.31; their OMR was 2.31 times their EMR. Given that the SOMR for pneumonia in 2013 in NYS was 4.60%, the RAMR for Ellenville Regional Hospital for pneumonia in 2013 was 2.31 * 4.60% = 10.65%.

Without further analysis, it is easy to interpret this hospital’s performance in pneumonia in 2013 as very poor. Based on its RAMR, if this hospital treated every pneumonia patient in NYS in 2013, then 10.65% of them would have died, not 4.60%; there would have been 2.31 times as many pneumonia deaths.

The NYS DOH^15^ computes a 95% CI for every RAMR, and in this case the interval is (1.29%, 38.45%). This is a very wide confidence interval primarily because the sample size is small. Also, since the CI for the RAMR contains the SOMR (4.60%), the NYS DOH reports that the difference between Ellenville Regional Hospital’s RAMR and the SOMR was not statistically significant.

Given that this information is made public, and given the public’s general lack of understanding of the proper interpretation of confidence intervals and statistical significance, Ellenville Regional Hospital runs the very real risk that people, including the media, will focus only on the RAMR since it is the performance measure that the State uses. The mysterious confidence interval and the reference to “not statistically significant” is likely to be ignored.

*The interpretation of the RAMR is obscure:* The NYS DOH web site that provides the data to the public includes the formula given above for computing the RAMR but we could find no explanation regarding its interpretation. One interpretation is that, if every patient in NYS with the given condition had been treated at the given hospital, then the RAMR represents the estimated proportion that would have died. We seriously doubt that many people would have come to this interpretation without guidance.

Moreover, even if they had, it is unlikely that they would have recognized the amount of sampling error in the RAMR, especially when the sample size is small. We can only wonder how many pneumonia (and other) patients might have boycotted Ellenville Regional Hospital because of its high RAMR, even though it was not statistically significantly different from the statewide rate.

*The normal distribution is not statistically justified in many cases:* The computations performed for NYS DOH by The Leapfrog Group^3^ analyze only situations in which the number of cases equals 30 or more. They state “This minimum reporting requirement was identified from the literature, which suggests that 30 cases is generally the point at which a non-normal distribution begins to approximate a *normal distribution*, which is important given the Safety Score’s use of z-scores for standardizing data across disparate data sets.”^3^

This is a misuse of the normal approximation. The rule cited refers to using the normal approximation to the sampling distribution of the *mean*. A mortality rate is a *proportion*, not a mean. The commonly used rule for using the normal approximation to the sampling distribution of the proportion is that both nπ ≥ 10 and n(1-π) ≥ 10, where π is the hospital’s EMR. For pneumonia in 2013, these requirements were *not met* in 110 of the 181 hospitals (60.8%) of the hospitals with 30 or more cases.

*CIs are often misunderstood:* The importance of avoiding confidence intervals should not be minimized. Several authors including Hoekstra et al,^18^ Belia et al,^19^ Gigerenzer,^20^ and Lecoutre et al^21^ have demonstrated the widespread inability of undergraduate and master’s degree students, researchers, and even statisticians to properly interpret confidence intervals and the associated null hypothesis significance tests. More relevant to the current application, Wulff et al^22^ showed the difficulty that physicians have in properly interpreting a wide range of statistical results, and Scheutz et al^23^ replicated those results for dentists. Given that professionals who use statistical inference regularly have problems with the proper interpretation of confidence intervals, we question the wisdom of using confidence intervals in a context in which the public is expected to use them in making critical healthcare decisions. Clearly the difficulty in the interpretation of statistical methods makes the interpretation of the quality reports confusing. In addition, Porter et al^24^ states “sensitivity to physicians’ concerns about being judged unfairly results in a tendency to exclude patients from outcomes comparisons instead of incorporating accepted risk-adjustment methods.”

*There is no adjustment for multiple comparisons:* NYS constructs confidence intervals for each RAMR and uses them to test the null hypothesis that the hospital’s performance does not differ from the statewide performance. We believe that the state should instead view this exercise as a screening process, not as a hypothesis testing process. There are two reasons why we advocate screening rather than hypothesis testing.

First, the state examines hundreds, if not thousands, of such analyses each year (many conditions across nearly 200 hospitals). This means that the state would need to use a multiple comparisons procedure, such as Bonferroni^25^ or Benjamini and Hochberg,^26^ which would be unlikely to serve the desired purposes in these circumstances. Second, there is no *a priori* theoretical reason to believe that any given hospital is either better or worse than average.

### Proposed Alternative to the Risk-Adjusted Mortality Rate

Our methodology uses two measures: the upper tail probability (UTP) to screen for hospitals performing poorly and the lower tail probability (LTP) to screen for hospitals performing well. Let *n* be the number of patients treated, *d* be the number of observed deaths, and *π* be the EMR. Then the UTP = P(X ≥ d | n, π) and the LTP = P(X ≤ d | n, π). It should be noted that both the UTP and the LTP include the number of deaths, *d*, so they do not sum to one.

The UTP computes the probability that the hospital would have had as many deaths as they did, or more, given their number of cases and their EMR. A small UTP indicates that the hospital’s number of deaths is unusually high. The LTP computes the probability that the hospital would have had as few deaths as they did, or fewer, given their number of cases and their EMR. A small LTP indicates that the hospital’s number of deaths is unusually low. Therefore, with either the UTP or the LTP, a small value represents either unusually poor performance or unusually good performance.

Hamm^27^ drew on the work of four previous studies to construct the list of verbal interpretations of probabilities shown in Table 1. For screening purposes, a UTP or an LTP less than 5% or 10% might be considered “very unlikely” or “rare” and therefore subject to further investigation. The choice, of course, depends on the decision-maker.

**Table 1:** List of Verbal Interpretations of Probabilities, From Table 1 of Hamm^27^

**Verbal Expression**	**Value**
Absolutely impossible	0.00
Rarely	0.05
Very unlikely	0.10
Seldom	0.15
Not very probable	0.20
Fairly unlikely	0.25
Somewhat unlikely	0.33
Uncertain	0.40
Slightly less than half of the time	0.45
Toss-up	0.50
Slightly more than half the time	0.55
Better than even	0.60
Rather likely	0.70
Good chance	0.75
Quite likely	0.80
Very probable	0.85
Highly probable	0.90
Almost certain	0.95
Absolutely certain	1.00

We approximate the UTP and the LTP using the binomial distribution function. We recognize that the use of the binomial distribution in this application technically requires that each patient in each hospital with a given condition must have the same probability of death, which is not the case since patients are known to have different patient and case characteristics. The calculation of the exact values of the UTP and the LTP would require the individual patient probabilities of death, which are not publicly available. If they were, the resulting probability distribution would be the so-called Poisson binomial distribution.

Under the Poisson binomial distribution, the expected number of deaths would equal the expected number under the traditional binomial distribution but the variance of the number of deaths would be smaller. Thus, the tail areas computed using the binomial are larger than those computed using the Poisson binomial (see Hoeffding^28^ and Boland^29^). Thus, the UTP and LTP values reported herein are conservatively large in the sense that any UTP or LTP that we report is greater than the values computed using the individual patient probabilities of death.

To illustrate the appropriateness of using the binomial distribution as an approximation when computing the UTP and LTP, we conducted a simple simulation with 100 patients. Suppose that the probabilities of death of the 100 patients, p_1_, p_2_, …, p_100_, are uniformly distributed between 0.01 and 0.09. We simulated the 100 probabilities of death and then simulated whether each patient lived or died to obtain the simulated total number of deaths. We repeated this process 1000 times to obtain the estimated probability distribution of the total number of deaths. From this distribution, we computed the UTP for each possible number of deaths; we call these the *actual UTPs*. Finally, we computed the *estimated UTPs* for each possible number of deaths using the binomial distribution with π = 0.05, the mean of the uniform distribution between 0.01 and 0.09.

**Figure 1:**
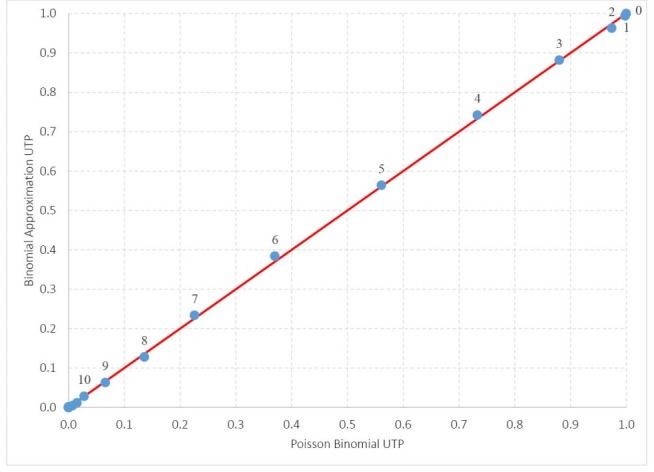
Binomial Approximation to the Poisson Binomial UTP for Each Number of Deaths. Abbreviation: UTP, upper tail probability.

Table 2 shows the actual and estimated UTPs. Figure 1 shows the relationship between these values. Clearly, the binomial distribution provides a very close approximation. The largest positive difference between the actual UTP minus the estimated UTP is 0.974–0.963 = 0.011, which occurs at 2 deaths, while the largest negative difference is 0.370–0.384 = −0.014, which occurs at 6 deaths. There is no apparent pattern in the differences between the actual and estimated UTPs.

**Table 2:** Simulated UTP Versus Binomial UTP in 1000 Simulation Replications

**Deaths**	**Simulated Frequency**	**Simulated Relative Frequency**	**Binomial Probability**	**Simulated UTP**	**Binomial UTP**	**Simulated UTP − Binomial UTP**
0	2	0.002	0.006	1.000	1.000	0.000
1	24	0.024	0.031	0.998	0.994	0.004
2	94	0.094	0.081	0.974	0.963	**0.011**
3	147	0.147	0.140	0.880	0.882	-0.002
4	172	0.172	0.178	0.733	0.742	-0.009
5	191	0.191	0.180	0.561	0.564	-0.003
6	144	0.144	0.150	0.370	0.384	**-0.014**
7	90	0.090	0.106	0.226	0.234	-0.008
8	70	0.070	0.065	0.136	0.128	0.008
9	38	0.038	0.035	0.066	0.063	0.003
10	13	0.013	0.017	0.028	0.028	0.000
11	8	0.008	0.007	0.015	0.011	0.004
12	7	0.007	0.003	0.007	0.004	0.003

‏ Abbreviation: UTP, upper tail probability.

Recall the case of Ellenville Regional Hospital, which, in 2013, had 2 deaths among 42 pneumonia patients whose EMR was 0.0206. Its RAMR was 10.65, or 2.31 times the SOMR. Recall also that it was only after considering the confidence interval that we could declare that its RAMR was not statistically different from the SOMR.

The UTP in this situation is the binomial probability P(X ≥ 2 deaths | n = 42, π = 0.0206), which equals 0.214. Thus, there is a 21.4% chance that Ellenville Regional Hospital would have experienced 2 or more pneumonia deaths given that the EMR of its 42 pneumonia patients was 2.06%. This is between “Not very probable” and “Fairly unlikely” on Hamm’s scale. It is clear that Ellenville Regional Hospital’s performance in pneumonia in 2013 did not approach the level of requiring greater scrutiny even though its RAMR was 2.31 times the SOMR. All that needs to be reported is that its UTP is 21.4% and its interpretation is clear: If Ellenville Regional Hospital treated 42 pneumonia patients with the same EMR every year, then it would experience two or more deaths in 21.4% of the years. There is no need for a confidence interval.

Next consider an imaginary Hospital A that treated 420 pneumonia cases and experienced 20 deaths. Suppose also that its EMR was 2.06%. Then its OMR would equal 20/420 = 4.76%, and its RAMR would be 10.65, all equal to Ellenville’s values. However, its UTP would be P(X ≥ 20 deaths | n = 420, π = 0.0206) = 0.00057. This is two orders of magnitude below “Rarely” and is very close to “Absolutely impossible.”

How often does an event with probability 0.00057 occur? The geometric distribution with *P* = .00057 has an expected value of 1/0.00057 = 1754, meaning that, if Hospital A treated 420 pneumonia patients with the same EMR every year, then it would experience 20 or more deaths on average once every 1754 years. The observed number of deaths at Hospital A should therefore be considered highly unusual while Ellenville’s is not unusual at all. This demonstrates that the RAMR is insensitive to case volume, which is incorporated naturally into the UTP in a statistically sound manner.

Note that the normal approximation is not needed to compute the UTP and the LTP. Therefore, we can analyze hospitals regardless of the number of cases they treated and their EMRs. Thus, by switching to the UTP/LTP methodology, no such methodological restrictions exist; NYS could assess situations with any number of cases.

## Results

We demonstrate our methodology using data from the NYS DOH web site.^15^ The database contains the number of cases treated, the number of deaths, and the EMRs for each of 10 IQIs for the years 2009-2013. The risk adjustments for the IQIs studied are explained in the AHRQ quality indicators, IQI parameter estimates.^24^ NYS reports this data only for hospitals that had 30 or more cases in the given IQI in the given year.

### Comparison of the Upper Tail Probability and the Risk-Adjusted Mortality Rate

We computed the 5-year overall RAMR and UTP across all IQIs for 196 hospitals. The Spearman rank correlation is −0.8559 (*P* < .00005), demonstrating strong agreement between the two measures. This supports our contention that the UTP is a viable alternative to the RAMR.

### The RAMR is Not Currently Applied When the Number of Cases is Less Than 30

For any situation with fewer than 30 cases, NYS does not report the number of cases, the number of deaths, or the EMR. However, the State does report the statewide total for cases and deaths, which allows us to compute the numbers of cases and deaths for situations with less than 30 cases taken collectively. In Table 3, we show that the unadjusted odds ratios of mortality for patients treated in hospitals that perform fewer than 30 cases per year ranges between 1.37 and 3.71 relative to patients treated in hospitals that perform more than 30 cases per year.

**Table 3:** Odds Ratio of Death in Each of 10 IQIs for Hospitals With Fewer Than 30 Cases Versus Hospitals With 30 or More Cases

**IQI Code**	**IQI Value**	**Volume**	**Cases**	**Deaths**	**OMR (%)**	**Odds**	**Odds Ratio**	**% Cases**	**% Deaths**
IQI08	Esophageal resection mortality rate	<30	189	7	3.70	0.0385	1.625	52.2	63.6
		≥30	173	4	2.31	0.0237		47.8	36.4
IQI09	Pancreatic resection mortality rate	<30	316	17	5.38	0.0569	3.582	23.6	51.5
		≥30	1024	16	1.56	0.0159		76.4	48.5
IQI11	AAA repair mortality rate	<30	713	42	5.89	0.0626	1.834	31.6	45.2
		≥30	1545	51	3.30	0.0341		68.4	54.8
IQI13	Craniotomy mortality rate	<30	571	85	14.89	0.1749	3.708	5.9	17.1
		≥30	9168	413	4.50	0.0472		94.1	82.9
IQI15	AMI mortality rate	<30	742	86	11.59	0.1311	1.944	2.5	4.5
		≥30	28 945	1829	6.32	0.0675		97.5	95.5
IQI16	Heart failure mortality rate	<30	211	16	7.58	0.0821	1.867	0.4	0.6
		≥30	58 635	2469	4.21	0.0440		99.6	99.4
IQI18	Gastrointestinal hemorrhage mortality rate	<30	251	14	5.58	0.0591	2.048	0.8	1.6
		≥30	30 821	864	2.80	0.0288		99.2	98.4
IQI19	Hip fracture mortality rate	<30	625	24	3.84	0.0399	1.374	4.4	5.9
		≥30	13 559	383	2.82	0.0291		95.6	94.1
IQI20	Pneumonia mortality rate	<30	151	12	7.95	0.0863	1.792	0.3	0.6
		≥30	45 387	2086	4.60	0.0482		99.7	99.4
IQI31	Carotid endarterectomy mortality rate	<30	564	3	0.53	0.0053	1.505	13.3	18.8
		≥30	3671	13	0.35	0.0036		86.7	81.3
	Combined	<30	4333	306	7.06	0.0760	1.728	2.2	3.6
		≥30	192 928	8128	4.21	0.0440		97.8	96.4

Abbreviations: UTP, upper tail probability; AAA, abdominal aortic aneurysm; AMI, acute myocardial infarction; IQI, inpatient quality indicator; OMR, observed mortality rate.

It is possible that the adjustment for EMR, for which the data are unavailable, would explain this difference. This might happen if those situations involving fewer than 30 cases also had higher EMRs relative to patients treated in situations involving 30 or more cases. To check this possibility, we fit a linear regression model using EMR (computed for each hospital across all IQIs and all years) as the dependent variable and the number of cases (over all IQIs and years) as the independent variable. The resulting model, with n = 196 hospitals, yields a highly statistically significant positive slope (*P* < .00005) suggesting that hospitals with fewer than 30 cases in an IQI are unlikely to have patients that are, on average, at higher risk of dying.

While it is true that only 2.2% of the cases in the 10 IQIs were treated in settings with fewer than 30 cases, in some IQIs, the percentage is much higher: 52.2% for esophageal resection, 31.6% for abdominal aortic aneurysm (AAA) repair, 23.6% for pancreatic resection and 13.3% for carotid endarterectomy.

The State does not evaluate these situations, apparently due to its reliance on the normal approximation to the binomial for the purpose of computing a confidence interval for the RAMR. By switching to the UTP/LTP methodology, no such methodological restrictions exist; the State could assess situations with any number of cases.

### Screening Rather Than Classification

The UTP and the LTP are not to be confused with upper-tail and lower-tail *P* values commonly used in hypothesis testing. There are no hypotheses here to test. We do not, for example, create a null hypothesis that the hospital’s mortality rate is greater (less) than or equal to its EMR and then use the UTP (LTP) to reject or not reject this null hypothesis. To do so would require solution of the massive multiple comparisons problem^25,26^ resulting from the very large number of hypothesis tests being performed across all hospitals, all diseases and conditions, and all years. Rather, the purpose of the analysis is to screen for circumstances in which there may have been more (fewer) deaths than might be expected based on their number of cases and EMR.

**Figure 2:**
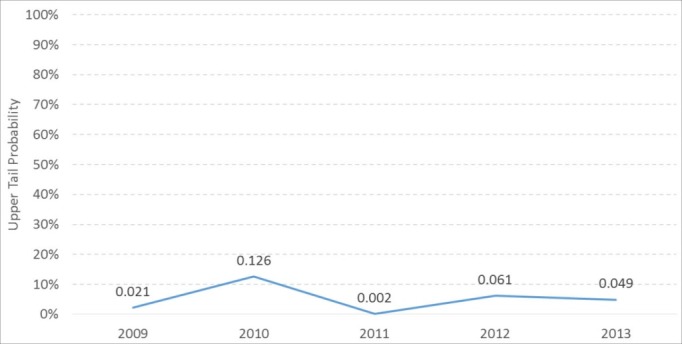
The UTPs for Heart Failure Mortality Rate in Hospital M Between 2009 and 2013. Abbreviation: UTP, upper tail probability.

Rather than classify a hospital’s performance as average, below average, or above average, which would imply a hypothesis testing framework, we propose using the UTP as a screening measure to identify situations in which there are likely to be opportunities for improvement. For example, Table 4 shows the performance of Hospital M. While Hospital M did very well overall and in three of the nine IQIs, its UTP in one IQI (heart failure mortality rate) is small (0.049). It is instructive to examine Hospital M’s UTP for the same IQI over the four previous years, as shown in Figure 2. It is clear that Hospital M’s mortality rate performance with respect to heart failure mortality rate has been low for at least 5 years.

**Table 4:** UTPs and LTPs for Hospital M for all IQIs Combined and Individually in 2013

**IQI**	**UTP**	**LTP**	**Cases**	**Deaths**	**OMR (%)**	**EMR (%)**
All IQIs Combined	0.995	0.007	3722	95	2.55	3.26
AAA repair mortality rate	1.000	0.048	107	0	0.00	2.80
AMI mortality rate	1.000	0.000	655	14	2.14	4.98
Carotid endarterectomy mortality rate	1.000	0.837	66	0	0.00	0.27
Craniotomy mortality rate	0.999	0.002	435	10	2.30	5.17
Gastrointestinal hemorrhage mortality rate	0.622	0.508	462	9	1.95	2.08
Heart failure mortality rate	0.049	0.967	1034	37	3.58	2.67
Hip fracture mortality rate	0.248	0.878	134	5	3.73	2.51
Hip replacement mortality rate	0.301	0.949	237	1	0.42	0.15
Pancreatic resection mortality rate	0.920	0.285	74	1	1.35	3.36
Pneumonia mortality rate	0.680	0.408	518	18	3.47	3.80

Abbreviations: UTP, upper tail probability; AAA, abdominal aortic aneurysm; AMI, acute myocardial infarction; IQI, inpatient quality indicator; OMR, observed mortality rate; EMR, expected mortality rate; UTP, upper tail probability; LTP, lower tail probability.

## Discussion

In this paper, we make two essential points. First, measuring hospital performance is essential for quality improvement but hypothesis testing is not the appropriate approach. Rather, agencies should take a screening approach that seeks to identify both where attention may be needed to improve performance, and where superior performance is occurring that might be emulated elsewhere. Second, the current RAMR methodology should be replaced. Our proposed alternative method, using the UTP and the LTP, should be considered as a replacement.

Briefly, we have shown that:

1. The RAMR does not provide the information necessary to determine whether a hospital’s performance is especially bad or especially good, in part because it is insensitive to sample size;
2. The interpretation of the RAMR is obscure to many people;
3. The use of the normal distribution to construct a confidence interval for the RAMR is not statistically justified in many cases;
4. While a proper confidence interval for the RAMR can be constructed without reference to the normal distribution, there is considerable evidence that large portions of the population, including physicians, other healthcare professionals, and the public, do not have a sufficiently fundamental understanding of confidence intervals to use them as a basis for healthcare decision-making; and
5. The RAMR and its confidence interval are portrayed as if a two-tailed hypothesis test were being performed without any attempt to adjust for the multiple comparisons that are being made.

The current implementation of the RAMR in NYS does not examine situations in which a hospital has treated fewer than 30 cases in a given IQI. We have found that such situations may account for as much as half of all such cases statewide. This failure is particularly troubling in light of the suggestive evidence that higher volume providers tend to perform better. This shortcoming disappears when using the UTP/LTP approach.

For the purposes of our study, we were unable to apply our methodology to situations with fewer than 30 cases since NYS DOH does not publish the necessary data. However, there is no reason to suspect that there would be any problems computing the UTP since the binomial distribution is readily available for any number of trials.

Our planned future research involves comparing the UTP/LTP results with the quality report cards prepared for each hospital. To the degree to which they agree, we will know that the UTP/LTP and the report cards are sensing similar phenomena. To the extent that they disagree, we will have an opportunity to learn more about the hospital’s quality performance.

In summary, our proposed method is simpler to implement than the RAMR – no need for a confidence interval – and it is easy to interpret. It is statistically sound and is applicable to all situations regardless of the number of cases treated, therefore providing a more comprehensive assessment of performance.

## Ethical issues

Not applicable.

## Competing interests

Authors declare that they have no competing interests.

## Authors’ contributions

CP’s contributions come from her expertise and experience in hospital management. TRS’s contributions arise from his education and experience in statistics and mathematical modeling.

## Key messages

**Implications for policy makers**

Policy-makers can benefit from the results of our study in the following ways:

- We frame the evaluation of hospitals in terms of screening rather than hypothesis testing.
- Unlike the risk-adjusted mortality rate (RAMR) , our method can be applied to all situations, regardless of the number of cases, thereby providing a more comprehensive evaluation of each hospital.
- Our alternative measure has a clear and practical interpretation.

**Implications for public**

In choosing a hospital for treatment of a serious disease or condition, you will want to consider many factors, perhaps the most important being your likelihood of surviving the hospitalization. You may find the risk-adjusted mortality rate (RAMR) difficult to interpret since that will require that you have a benchmark value for comparison and an understanding of confidence intervals. Our proposed method will provide you with a measure that:

- has a clear and practical interpretation,
- does not rely on a benchmark or an understanding of confidence intervals, and
- is available regardless of the number of cases were treated by the hospital last year. Therefore, you will be sure to have information on all hospitals under consideration, and that the information will be readily understood without confusion.
